# Advanced practice provider angle: American Society for Gastrointestinal Endoscopy advanced practice provider case of the month

**DOI:** 10.1016/j.igie.2024.07.002

**Published:** 2024-07-23

**Authors:** Stacia Sackmaster, Sarah Enslin, Joseph Vicari

**Affiliations:** 1 Rockford Gastroenterology Associates, Rockford, Illinois, USA; 2 Department of Gastroenterology and Hepatology, University of Rochester Medical Center, Rochester, New York, USA

## Case 17: General Gastroenterology

A 65-year-old woman was hospitalized after a right hip replacement 3 days before. A gastroenterology consult was requested for evaluation of abdominal distention. She had poor oral intake and was receiving regularly scheduled intravenous morphine for hip pain. She had been immobile for the last 2 days and the day before noted abdominal distention. Her last bowel movement was 3 to 4 days ago, and she last passed flatus 1 day ago. The complete blood count and electrolytes were normal. Abdominal films reveal a dilated right-sided colon, air in the rectum, and normal small bowel. A CT of the abdomen and pelvis revealed proximal colonic dilation to 8 cm, normal to mildly dilated left-sided colon, air in the rectum, and no obstruction.

The most likely diagnosis is

A. Acute colonic pseudo-obstruction (Ogilvie’s syndrome)
B. Mechanical colonic obstruction
C. Small-bowel obstruction
D. Small-bowel ileus
E. Gastroparesis

The correct answer is A, acute colonic pseudo-obstruction (Ogilvie’s syndrome).

## Practice Pearls

### Epidemiology

• Acute colonic pseudo-obstruction typically involves the cecum and right-sided colon, occasionally extending to the rectum.^1^
• Acute colonic pseudo-obstruction most often occurs in patients with severe illness or injury and with recent surgery including abdominal surgery, heart surgery, and especially knee and hip surgery.^1^^,^^2^ Additional factors include medications (eg, opiates, anticholinergics, and calcium channel blockers), neurologic disorders, and electrolyte abnormalities.^1^^,^^2^
• The precise mechanism by which colonic dilation occurs is unknown.^1^

### Clinical manifestations

• Clinical manifestations include abdominal distension (most common symptom), abdominal pain, nausea, vomiting, constipation, and paradoxical diarrhea.^1^^,^^2^ Nearly half of patients with Ogilvie’s syndrome report passing flatus.
• Abdominal distension occurs gradually over 3 to 7 days; however, symptoms can begin as early as 24 to 48 hours.^1^
• On examination, the abdomen is tympanic to percussion. Typically, bowel sounds are present. Approximately 65% of patients have abdominal tenderness.^1^^,^^2^ The presence of fever, marked abdominal tenderness, and peritoneal signs suggests colonic ischemia or perforation.^1^

### Diagnostic approach

**Figure 1:**
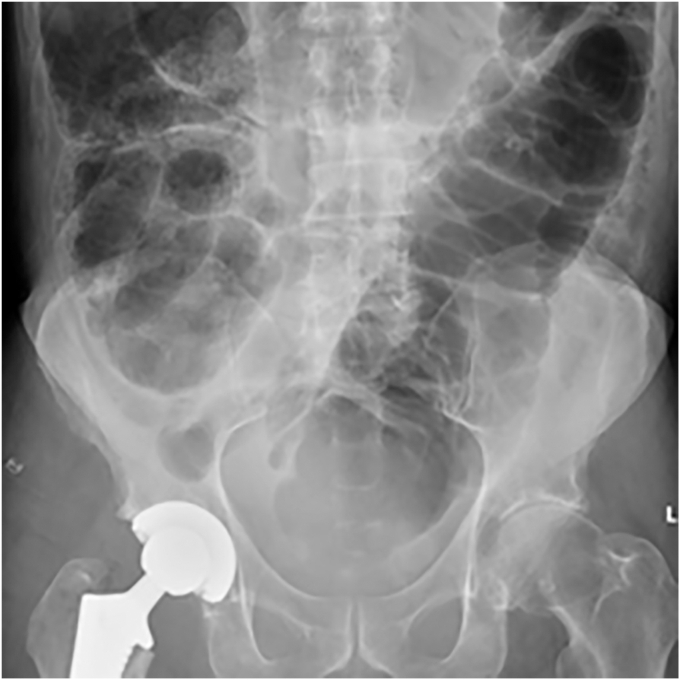
Abdominal film showing right-sided colon distension with air in the rectum.
• CT of the abdomen and pelvis reveals proximal colonic dilation that may extend to the rectum while ruling out colonic obstruction. When a CT is not available, a contrast enema using water-soluble contrast can be used, providing there is no evidence of perforation on examination.^1^

• Laboratory evaluation includes a complete blood count, comprehensive metabolic panel, serum lactate, and thyroid-stimulating hormone.
• Abdominal films typically reveal right-sided colonic distension with air in the rectum (Fig. 1).

### Management when cecal diameter is <12 cm

• Initial management is conservative with supportive care: treat the underlying cause, discontinue medications associated with acute colonic pseudo-obstruction, nasogastric tube decompression and cautious administration of tap water enemas.
• Patients should be monitored with physical examination every 12 to 24 hours, with plain abdominal films taken every 12 to 24 hours as well as laboratory values including complete blood count and comprehensive metabolic panel.^1^

### Management if cecal diameter is >12 cm, severe abdominal pain, or failed conservative management

• Neostigmine is the recommended initial treatment provided there are no contraindications to neostigmine (eg, recent myocardial infarction, asthma, bradycardia, or therapy with beta-blockers).^1^ Neostigmine is administered by slow intravenous infusion over 5 minutes. Patients should be kept supine on a bedpan, atropine should be available at the bedside, along with continuous monitoring of vital signs and electrocardiogram for 30 minutes, and continuous clinical assessment for 15 to 30 minutes.^1^^,^^3^
• Adverse events of neostigmine include bradycardia, bronchoconstriction, hypotension, and abdominal cramps.
• Response to therapy with resolution of acute colonic pseudo-obstruction occurs in up to 89% of patients. Patients who fail to respond to the first dose of neostigmine can receive a second dose 24 hours after the first dose.^1^^,^^4^^,^^5^
• Colonic decompression is performed in patients who failed therapy with neostigmine or who have contraindications to neostigmine. Colonic decompression is a technically difficult procedure to perform and has a perforation rate of approximately 2%.^1^
• A decompression tube, positioned over a guidewire, can be placed during colonic decompression with colonoscopy.
• Surgical management is reserved for patients with refractory symptoms, ischemia, perforation, or peritonitis.

## Patient consent

This case was developed based on the signs and symptoms observed in various patients.Â Patient consent was received.

## Disclosure

All authors disclosed no financial relationships.
